# A Mitogenomic Phylogeny of Living Primates

**DOI:** 10.1371/journal.pone.0069504

**Published:** 2013-07-16

**Authors:** Knut Finstermeier, Dietmar Zinner, Markus Brameier, Matthias Meyer, Eva Kreuz, Michael Hofreiter, Christian Roos

**Affiliations:** 1 Research Group Molecular Ecology, Max Planck Institute for Evolutionary Anthropology, Leipzig, Germany; 2 Cognitive Ethology Laboratory, German Primate Center, Leibniz Institute for Primate Research, Göttingen, Germany; 3 Primate Genetics Laboratory, German Primate Center, Leibniz Institute for Primate Research, Göttingen, Germany; 4 Gene Bank of Primates, German Primate Center, Leibniz Institute for Primate Research, Göttingen, Germany; University of Florence, Italy

## Abstract

Primates, the mammalian order including our own species, comprise 480 species in 78 genera. Thus, they represent the third largest of the 18 orders of eutherian mammals. Although recent phylogenetic studies on primates are increasingly built on molecular datasets, most of these studies have focused on taxonomic subgroups within the order. Complete mitochondrial (mt) genomes have proven to be extremely useful in deciphering within-order relationships even up to deep nodes. Using 454 sequencing, we sequenced 32 new complete mt genomes adding 20 previously not represented genera to the phylogenetic reconstruction of the primate tree. With 13 new sequences, the number of complete mt genomes within the parvorder Platyrrhini was widely extended, resulting in a largely resolved branching pattern among New World monkey families. We added 10 new Strepsirrhini mt genomes to the 15 previously available ones, thus almost doubling the number of mt genomes within this clade. Our data allow precise date estimates of all nodes and offer new insights into primate evolution. One major result is a relatively young date for the most recent common ancestor of all living primates which was estimated to 66-69 million years ago, suggesting that the divergence of extant primates started close to the K/T-boundary. Although some relationships remain unclear, the large number of mt genomes used allowed us to reconstruct a robust primate phylogeny which is largely in agreement with previous publications. Finally, we show that mt genomes are a useful tool for resolving primate phylogenetic relationships on various taxonomic levels.

## Introduction

An accurate and reliable phylogeny provides information about evolutionary relationships among species and higher taxa, and can be used to determine the timescale of their evolution. Thus, phylogenetic reconstructions serve as a basis for comparative analyses of adaptive processes and for the discrimination between ancestral and derived states (e.g. [1–4]). The use of sequence data and other genetic markers has strongly improved phylogenetic reconstructions. Depending on the mode of inheritance of the respective marker used (autosomal, Y chromosomal or mitochondrial), different questions concerning a phylogeny can be resolved. In animals, the mitochondrial (mt) genome is typically maternally inherited, non-recombining, and has a relatively high substitution rate and a smaller effective population size than the nuclear genome [5–9]. These properties can increase the probability of congruence between the mitochondrial gene tree and the species tree, helping to resolve relationships between recently divergent taxa [10], in particular if complete mt genome information is used instead of single gene information [11–14].

Although various studies on primate phylogeny combining mtDNA and nuclear DNA fragments in supermatrix approaches [15–17] or relying solely on nuclear DNA [18] were recently published, the molecular phylogeny of primates is still incompletely resolved and particularly a comprehensive phylogeny based on mt genomes alone is not yet available. Previous phylogenetic studies using only mitochondrial markers have mainly used fragments of the mt genome or, if sequence information of the complete mt genome was used, these studies either included only a few species (e.g., [19–21]) or focused on certain taxonomic groups within the primate order (e.g., strepsirrhines [22],; platyrrhines [23],; colobines [24–27],; gibbons [28,29],; chimpanzees [1],; and humans [30],). A generic mitochondrial phylogeny based on complete mt genome information is still lacking for primates. To overcome this limitation, we generated 32 complete primate mt genomes using next-generation-sequencing and combined them with 51 additional mt genome sequences from GenBank to reconstruct a robust family-level phylogeny of primates and to estimate the respective divergence times using solely primate fossil calibration points.

## Results and Discussion

We produced complete mt genome sequences from 32 primate individuals. From each individual, we obtained an average of 1508 tagged reads with an average length of 235 bp, yielding approximately 356 kb of sequence data corresponding to 21-fold coverage. All newly sequenced mt genomes had lengths typical for primates (16,280–16,936 bp; Table S1), but the GC-content varied largely among taxa (37.78–46.32%, Table S2, Figure S1). All newly generated mt genomes consisted of 22 tRNA genes, 2 rRNA genes, 13 protein-coding genes and the control region in the order typical for mammals. By combining the 32 newly generated data with 51 additional primate mt genomes, the dataset represents all 16 primate families, 57 of the 78 recognized genera and 78 of the 480 currently recognized species [31].

**Figure 1:**
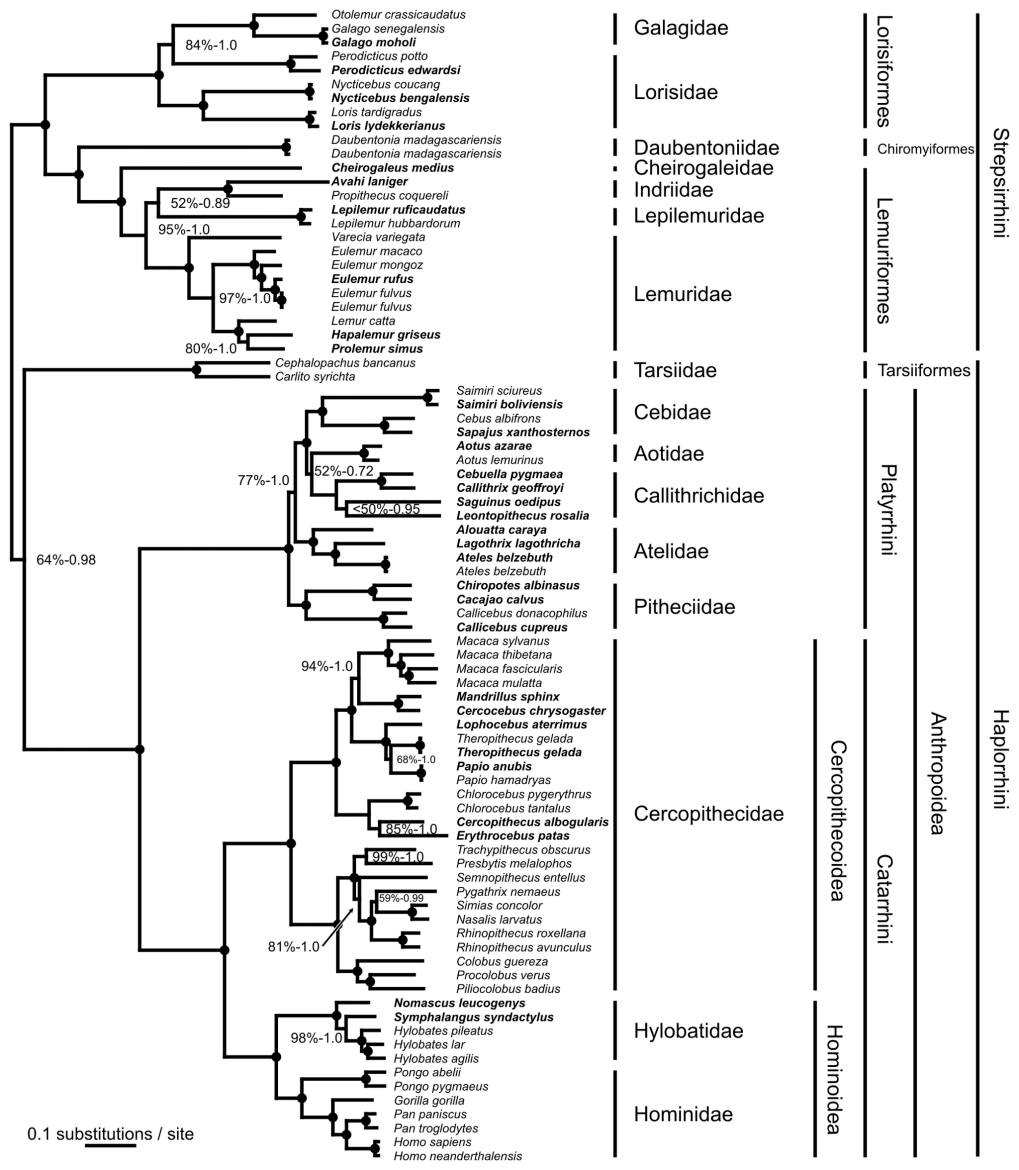
Phylogram showing the phylogenetic relationships among the investigated primate mt genomes as obtained from dataset mtDNA1. Newly generated sequences are indicated in bold. Black dots on nodes indicate ML support and Bayesian posterior probabilities of 100% and 1.0, respectively. Lower values are shown at the respective branches.

The phylogenetic relationships as revealed by both applied algorithms (maximum-likelihood [ML] and Bayesian inference) and for the different datasets are identical and predominantly strongly supported (Figure 1, S2, S3, S4). Only a few nodes obtained less statistical support and for the AGY datasets (mtDNA2, mtDNA4) support values were generally lower than for the AGTC datasets (mtDNA1, mtDNA3). Moreover, the tree topology is highly congruent with the ones obtained from nuclear sequence data [18], the presence/absence pattern of retroposon elements [26,32–42], supermatrix approaches [15–17] and mt genome data [19–29], although a few cases of incongruence remain (see below).

**Figure 2:**
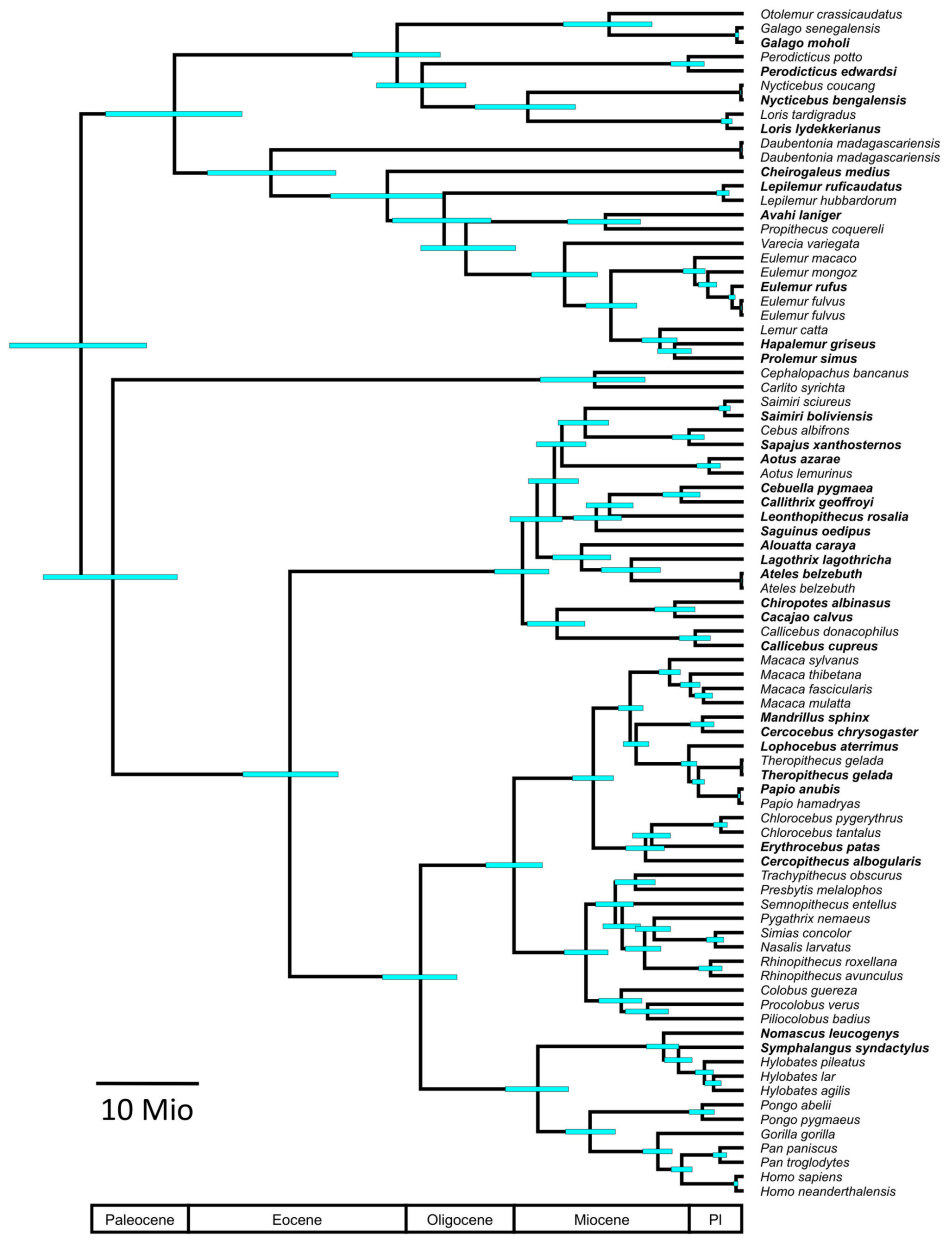
Estimated divergence ages as obtained from dataset mtDNA1 along with their 95% credibility intervals (blue bars). Newly generated sequences are indicated in bold. A geological time scale is given below the tree. For detailed information on estimated divergence ages see Table S3.

The estimated divergence ages from both AGCT datasets (mtDNA1, mtDNA3) are highly similar (Figure 2; Table S3). All estimates suggest that the most recent common ancestor (MRCA) of the primate order is more recent than suggested by various other genetic studies (e.g., [16,18,19,22,43–45], but see 15,46) and statistical modelling [47]. However, our estimate is in line with both fossil data [48,49], estimates based on expected life-history correlates of primates [50] and recent estimates based on a supermatrix approach [17].

### Early Primate Divergence

A longstanding problem in primate phylogeny and classification was the position of Tarsiiformes relative to Anthropoidea and Strepsirrhini [51]. Although only weakly supported in all our reconstructions, Tarsiiformes do always cluster together with Anthropoidea to the exclusion of Strepsirrhini. Accordingly, our findings are in agreement with retroposon integrations and nuclear sequence data [18,40] and support the initial primate divergence into Strepsirrhini versus Haplorrhini (Anthropoidea + Tarsiiformes). This initial split occurred 66.22-69.05 Ma (range of means from both estimates; for 95% credibility intervals see Table S3), suggesting a primate origin around the Cretaceous-Tertiary boundary [15,46]. Shortly afterwards, Tarsiiformes separated from Anthropoidea (63.07-64.81 Ma). Within Tarsiiformes, both analyzed genera, *Carlito* and *Cephalopachus*, diverged in the Middle Miocene, concordant with previous results [52].

### Strepsirrhini

Among strepsirrhines we found a division into the Malagasy lemurs (Chiromyiformes and Lemuriformes) and Lorisiformes, with both lineages separating in the Late Paleocene or Early Eocene (56.89-58.57 Ma). Within Lorisiformes, *Loris* *idae* appeared as a paraphyletic group with the African *Perodicticus* either forming a sister lineage to Galagidae or, to a clade containing Galagidae plus *Nycticebus* and *Loris*. However, support values for either branching pattern are low and a monophyletic *Loris* *idae* clade is statistically not rejected (P > 0.05, Table S4). Therefore, divergence age estimates are based on an a-priori constrained monophyly of *Loris* *idae* as suggested by retroposon integrations and nuclear sequence data [18,38]. According to this approach, both Lorisiformes families diverged around the Eocene-Oligocene boundary ca. 35 Ma, followed by a subsequent separation of African and Asian lorisids about 32 Ma. The genera within Galagidae and the Asian *Loris* *idae* emerged about 13 Ma and 21 Ma, respectively.

Malagasy lemurs appeared as a monophyletic clade with the basal split dating to about 47 Ma separating the Chiromyiformes ( *Daubentonia* *madagascariensis* ) from the Lemuriformes. As in earlier studies [15,16,18,38,53], the relationships among the four Lemuriformes families are not well resolved and various alternative relationships are not rejected. However, one retroposon integration supports an *Indri* *idae* + *Lemur* *idae* clade [38] and hence, both families were constrained to be monophyletic for divergence age estimations. Based on our estimates, lemuriform families emerged 26.52-35.47 Ma. Within *Indri* *idae* , the nocturnal *Avahi* and the diurnal *Propithecus* separated about 13 Ma. Within the family *Lemur* *idae* , *Varecia* diverged first (~ 17 Ma), while the remaining genera split into *Eulemur* and a clade consisting of *Lemur* , *Hapalemur* and *Prolemur* about 13 Ma. In the latter clade, *Lemur* appears as sister lineage to *Hapalemur* + *Prolemur*, suggesting a common origin of the bamboo lemurs ( *Hapalemur* and *Prolemur*). The branching of those three genera gained only weak statistical support and the divergence time estimates suggest a rapid divergence within a short time period during the Late Miocene.

### New World Monkeys (Platyrrhini)

We found Platyrrhini to have separated from Catarrhini about 46 Ma, which is in line with earlier studies [18,45,46,54,55]. Although only weakly supported, the branching pattern among platyrrhines with Pitheciidae diverging first and Atelidae forming a sister family to the remaining families is in agreement with various recent studies [16,18,23,36,37,56,57]. While Cebidae, Aotidae and Callithrichidae are strongly suggested as a monophyletic clade, the phylogenetic relationships among them remain unresolved and various alternative relationships are not rejected. According to our divergence age estimates, Pitheciidae split from other platyrrhine families about 22 Ma and further diverged into Callicebinae ( *Callicebus* ) and Pitheciinae ( *Cacajao* and *Chiropotes* ) about 18 Ma. Atelidae split from Cebidae, Aotidae and Callithrichidae about 20 Ma, while the latter three families originated during a short time period (18.03-18.97 Ma) in the Early Miocene. Further differentiation of families into subfamilies (Atelidae: Atelinae [*Ateles*, *Lagothrix* ] - Alouattinae [Alouatta]; Cebidae: Cebinae [*Cebus*, *Sapajus*] - Samirinae [Saimiri]) and initial splits within Callithrichidae occurred slightly later in the Middle Miocene. In Callithrichidae, the branching pattern among *Saguinus* , *Leontopithecus* and the *Callithrix* + *Cebuella* clade remains unresolved, but nuclear sequence data and retroposon integrations strongly suggest that the basal split separated *Saguinus* from all other lineages within the family; therefore, this branching pattern was fixed for divergence time estimations [18,36]. The genera within the different subfamilies originated during the Late Miocene ( *Chiropotes* - *Cacajao* , *Ateles* - *Lagothrix* , *Callithrix* - *Cebuella*) or Early Pliocene (*Cebus* - *Sapajus*).

### Old World Monkeys (Cercopithecoidea)

Old World monkeys separated from hominoids about 32 Ma, which is in broad agreement with earlier studies [18,22,45,54,55]. They further diverged into the subfamilies Cercopithecinae and Colobinae in the Early Miocene. During the Middle Miocene, Cercopithecinae further separated into Cercopithecini ( *Chlorocebus* *,* *Erythrocebus* *,* *Cercopithecus* ) and Papionini (*Macaca,* *Mandrillus* *,* *Cercocebus* *, Papio,* *Theropithecus* *,* *Lophocebus* ), while Colobinae diverged into the African Colobini (*Colobus,* *Procolobus* *, Piliocolobus*) and Asian Presbytini ( *Presbytis* *,* *Trachypithecus* *,* *Semnopithecus* *, Rhinopithecus,* *Pygathrix* *, Nasalis, Simias*). Interestingly, in both subfamilies we found several discordances between mt genome and nuclear data. In our mt genome data, *Erythrocebus* clusters with *Cercopithecus* and not with *Chlorocebus* as suggested by all available nuclear sequence and retroposon data [18,42,58,59], but monophyly of *Erythrocebus* and *Chlorocebus* is not rejected by alternative tree topology tests (P > 0.05). Thus, *Erythrocebus* and *Chlorocebus* were constrained in a monophyletic clade for calculating divergence ages, which resulted in estimates for the differentiation of Cercopithecini lineages between 8.88 and 9.59 Ma. In the Papionini, we found the *Mandrillus* + *Cercocebus* clade to be closer related to *Macaca* than to the other African genera (*Papio,* *Theropithecus* *,* *Lophocebus* ), which is in disagreement with nuclear sequence and retroposon data [18,41]. However, a sister position of *Macaca* to all other Papionini is not rejected and hence, for divergence age estimations, *Macaca* was constrained as sister group to all other members of Papionini. Accordingly, *Macaca* separated first (ca. 11.25 Ma), followed shortly afterwards by the separation of the *Mandrillus* + *Cercocebus* clade from the Papio + Theropithecus + *Lophocebus* clade about 10.5 Ma. The relationships within the latter clade are unresolved and suggest a rapid diversification about 5.2 Ma. In both the African and Asian colobines, the branching pattern among genera and respective divergence ages are similar to those found in other mt genome studies [26,27]. Similar to previous studies on colobines, our study provides evidence for a monophyletic odd-nosed monkey clade (*Rhinopithecus, Pygathrix, Nasalis, Simias*), which originated in the Late Miocene, but support for a monophyletic langur clade ( *Presbytis* *,* *Trachypithecus* *,* *Semnopithecus* ) [18,24,26,27,60,61] is missing. While nuclear sequence and retroposon data suggest a *Semnopithecus* + *Trachypithecus* clade [18,26,35,61], mt genome data indicate *Trachypithecus* to be related with *Presbytis* [26,27], thus supporting the hybridization scenario proposed by Roos et al. [26].

### Apes and Humans (Hominoidea)

In agreement with earlier studies [15–18,25,28,29,46,62–64], hominoids diverged into small apes or gibbons (Hylobatidae) and great apes and humans (Hominidae) in the Early Miocene. Within Hylobatidae, *Nomascus* separated first (ca. 7.8 Ma), followed by the divergence of *Symphalangus* and *Hylobates* ca. 6.2 Ma, which is concordant with other mtDNA data sets [28,29,64,65]. In concordance with other studies [15–18,22,25,45,66], in Hominidae, orang-utans ( *Pongo* ) diverged ca. 15.2 Ma from the African great apes and humans, while *Gorilla* separated from the Homo + *Pan* clade ca. 8.4 Ma. Finally, chimpanzees and humans separated in the Latest Miocene, about 5.9 Ma.

## Conclusions

Our study based on complete mt genomes of a large number of primates revealed a robust primate phylogeny with well-resolved phylogenetic relationships and predominantly strong node support. Moreover, the obtained phylogeny is largely in agreement with nuclear sequence and retroposon data, suggesting that the reconstructed relationships are indeed correct. However, there are some discordances between nuclear and mt genome phylogenies, some of which can be explained by hybridization and secondary gene flow, while for others, branching patterns as suggested by nuclear data cannot be excluded for the mt genome data. We also found that the observed shifts in G/C content among taxa have no major influence on the overall phylogeny. Interestingly, our estimate for the MRCA of all living primates dates to the Cretaceous-Tertiary boundary and thus much more recent than some other genetic studies have suggested. However, since we used only primate internal calibration points and since our estimates are in agreement with fossil data and expected life-history correlates of primates, we believe that this estimate is reliable. Overall, our study shows that complete mt genomes provide a better resolution of phylogenetic relationships on various taxonomic levels than short mt genome fragments or nuclear sequence data. Since hybridization among primate taxa is common [67], data from sex-specific inherited markers, i.e. mtDNA or Y-chromosomal loci is essential to trace such events and thus, our study will serve as basis for future studies on primate evolution and possible hybridization events.

## Ethical Statement

Samples were not specifically acquired for this study and all samples were provided by zoos in Amsterdam, Berlin, Cologne, Duisburg, Dresden, Gettorf, Mannheim, Munich, Romagne and Wuppertal, or by Prof Yves Rumpler. Most samples derived from zoo specimens. Respective samples were taken during routine veterinary care under general anaesthesia with a 2mg/kg injection of ketamine solution. Skin biopsies from *Avahi* *laniger* and *Lepilemur* *ruficaudatus* were obtained from wild animals, which were already used in earlier molecular studies ( [68,69]). Permission for field work and biopsy collection was provided by the Direction des Eaux et Forêts of Antananarivo and the Association Nationale pour la Gestion des Aires Protégées of Antananarivo to Prof Yves Rumpler. Sample collection was approved by the Animal Welfare Body of the German Primate Center and adhered to the American Society of Primatologists (ASP) Principles for the Ethical Treatment of Non-Human Primates (see www.asp.org/society/policy.cfm). No animals were sacrificed for this study.

## Materials and Methods

Primate DNA samples were obtained from the long-term collections of the authors or from colleagues (see Table S1 for a full list of samples). Two overlapping PCR fragments with sizes of 8 kb (primers 5’-GGCTTTCTCAACTTTTAAAGGATA-3’; 5’-TGTCCTGATCCAACATCGAG-3’) and 10 kb (primers 5’-CCGTGCAAAGGTAGCATAATC-3’; 5’-TTACTTTTATTTGGAGTTGCACCA-3’), respectively, that cover the entire mt genome were amplified using the Expand Long Range dNTPack (Roche). Initial denaturation was at 92° C for 2’, followed by (92° C for 10″, 60° C for 15″, 68° C for 8’) for 10 cycles, (92° C for 10″, 60° C for 15″, 68° C for 8’+20”/cycle) for 25 cycles, and a final extension at 68° C for 7’. After SPRI bead purification (AMPure, Beckman Coulter), PCR products were quantified on a Nanodrop and PCR products from identical samples were pooled in equimolar ratios. PCR products were then converted into bar-coded 454 sequencing libraries according to the Parallel-Tagged-Sequencing protocol [70]. Final library quantification was done via qPCR [71]. Pooled DNA libraries were sequenced on the 454 Flx Sequencing platform (Roche). Sequencing reads were sorted according to their molecular bar code using the program Untag [70] and assembled via the Newbler assembly program of the Roche 454 software tools. The consensus sequence of each mt genome was built on a >50%-majority rule. Gaps in genomic sequences and regions below 3-fold coverage were re-sequenced from shorter PCR fragments using Sanger sequencing. Mitochondrial genomes were annotated automatically using the DOGMA annotation software [72]. If the reading frame of protein coding genes was disrupted due to homopolymer length misidentification by the 454 post-processing software, the original read assembly was revised and corrected manually. All 454 sequences are available at the European Nucleotide Archive under study accession number ERP002564. Accession numbers for sample specific reads are given in Table S1. All assembled and annotated primate mt genomes are available at GenBank, accession numbers are given in Table S1.

To expand the dataset, we added 51 additional primate mt genomes available from GenBank as well as four non-primate mt genomes used as outgroups (Table S1). Data from GenBank were selected to represent complete mt genomes with no more than 10 ambiguous sites. Accordingly, the final dataset consisted of 87 mt genomes. An alignment was generated using MAFFT v 6.708b [73] with default settings (Data S1). Four different datasets were generated for phylogenetic reconstructions. For Dataset 1 (mtDNA1), poorly aligned positions and indels were removed with Gblocks v 0.91b [74] using default settings, and also the D-loop region was excluded (total length: 13,281 bp). Due to extreme shifts in C/T content among taxa as calculated in PAUP v4.0b10 [75] (Table S2, Figure S1), positions with C and T were replaced with Y (AGY) in the second dataset (mtDNA2). Dataset 3 (mtDNA3) and 4 (mtDNA4) were generated in Mesquite v 2.75 [76] and consisted only of the 12 protein-coding genes on the heavy strand (total length: 10,773 bp). In mtDNA4, C and T were again replaced with Y. Phylogenetic trees were constructed with ML and Bayesian algorithms, using the programs GARLI 2.0 [77] and MrBayes 3.1.2 [78,79]. For all reconstructions, the optimal nucleotide substitution model for each locus was chosen using the Bayesian information criterion (BIC) as implemented in jModeltest 2.1 [80]. For phylogenetic analyses, the datasets were whenever appropriate partitioned treating each locus separately and each with its own substitution model. In GARLI, only the model specification settings were adjusted, while all other settings were left at their default value. Relative support of internal nodes was assessed by bootstrap analyses with 500 replications and ML majority-rule consensus trees were calculated in PAUP. For Bayesian analyses, we used four independent Markov Chain Monte Carlo (MCMC) runs with the default temperature of 0.2. Four repetitions were run for 10 million generations with tree and parameter sampling occurring every 100 generations. Acceptance rates were in the optimal range of 10-70%. The first 25% of samples were discarded as burn-in, leaving 75,001 trees per run. The adequacy of this burn-in and convergence of all parameters was assessed by examining the uncorrected potential scale reduction factor (PSRF) [81] as calculated by MrBayes, which should approach 1 as runs converge and by visual inspection of the trace of the parameters across generations using the software TRACER 1.5 [82]. AWTY [83] was used to check whether posterior clade probabilities were also converging. Posterior probabilities for each split and a phylogram with mean branch lengths were calculated from the posterior density of trees. For the mtDNA1 dataset, various alternative phylogenetic relationships were tested with the Kishino-Hasegawa [84] and Shimodaira-Hasegawa [85] tests with full optimization and 1,000 bootstrap replications in PAUP.

To estimate divergence ages from datasets mtDNA1 and mtDNA3 we applied a Bayesian MCMC method, which employs a relaxed molecular clock approach [86] as implemented in BEAST 1.6.1 [87]. Therefore, we assumed a relaxed uncorrelated lognormal model of lineage variation and a Birth-Death Process prior for branching rates. Dataset mtDNA3 was further partitioned into codon positions and the substitution model, rate heterogeneity and base frequencies were unlinked across codon positions (1–3). Because some depicted branching patterns were only weakly supported or contradicted the nuclear phylogeny [18], these relationships were constrained if respective alternative relationships were not rejected by alternative tree topology tests (Table S4). Four replicates were run for 25 million generations with tree and parameter sampling occurring every 100 generations. The adequacy of a 10% burn-in and convergence of all parameters were assessed by visual inspection of the trace of the parameters across generations using TRACER v 1.5 [82]. Subsequently, the sampling distributions were combined (25% burn-in) using the software LogCombiner v 1.6.1 and a consensus chronogram with node height distribution was generated and visualized with TreeAnnotator v 1.6.1 and FigTree v 1.3.1 [88].

As calibration points, we used the same as in Perelman et al. [18] (Table S5): MRCA of Lorisiformes 40 Ma (SD = 3.0) [89], MRCA of Anthropoidea 43 Ma (SD = 4.5) [90,91], MRCA of Catarrhini 29.0 Ma (SD = 6.0) [91,92], MRCA of Platyrrhini 23.5 Ma (SD = 3.0) [23,93], MRCA of Papionini 7.0 Ma (SD = 1.0) [94], MRCA of *Theropithecus* and *Papio* 4.0 Ma (SD = 0.4) [58,60], MRCA of Hominidae 15.5 Ma (SD = 2.5) [22], MRCA of *Homo* and *Pan* 6.5 Ma (SD = 0.8) [95], and a primate MRCA of 90.0 Ma (SD = 6.0) [19,45,91]. All calibration points were applied as normal priors.

## Supporting Information

Table S1 Information on the studied species including mt genome length along with accession numbers for GenBank and the European Nucleotide Archive.
(XLS)

Table S2 Base composition of individual mt genomes and average base composition for the studied genera (in bold).
(XLS)

Table S3 Estimated divergence ages and 95% credibility intervals (in parentheses) for datasets mtDNA1 and mtDNA3 based on 9 internal calibration points and comparable estimates from earlier studies ( [15,17,18]).
(XLS)

Table S4 Results from alternative tree topology (Kishino-Hasegawa and Shimodaira-Hasegawa) tests for questionable relationships based on 1000 bootstraps. Shown are likelihoods and differences to the most probable topology. Significant (P<0.05) results are labeled with an asterisk.
(DOC)

Table S5 Calibration points used for divergence time estimates.
(XLS)

Figure S1 Diagram showing the G/C content of the mt genomes of the studied genera.
(TIF)

Figure S2 Phylogram as obtained from dataset mtDNA2. Newly generated sequences are indicated in bold. Black dots on nodes indicate ML support and Bayesian posterior probabilities of 100% and 1.0, respectively. Values below are shown at the respective branches.
(TIF)

Figure S3 Phylogram as obtained from dataset mtDNA3. Newly generated sequences are indicated in bold. Black dots on nodes indicate ML support and Bayesian posterior probabilities of 100% and 1.0, respectively. Values below are shown at the respective branches.
(TIF)

Figure S4 Phylogram as obtained from dataset mtDNA4. Newly generated sequences are indicated in bold. Black dots on nodes indicate ML support and Bayesian posterior probabilities of 100% and 1.0, respectively. Values below are shown at the respective branches.
(TIF)

Data S1 Original alignment of the 83 studied primate individuals and four outgroup taxa.
(FA) Click here for additional data file.
