# Supplementary material for: A Mitogenomic Phylogeny of Living Primates
Source: PLoS One. 2013 Jul 16;8(7):e69504. doi: 10.1371/journal.pone.0069504 (PMC3713065; doi:10.1371/journal.pone.0069504)
Supplement: Table S4 — Results from alternative tree topology (Kishino-Hasegawa and Shimodaira-Hasegawa) tests for questionable relationships based on 1000 bootstraps. Shown are likelihoods and differences to the most probable topology. Significant (P<0.05) results are labeled with an asterisk. (DOC) [file pone.0069504.s004.doc]

Table S3: Alternative tree topology tests

|  | **-ln L** | **difference** | **KH test** | **SH test** |
| --- | --- | --- | --- | --- |
| **Alternative positions of Tarsiidae** |  |  |  |  |
| ((Anthropoidea,Tarsiidae),Strepsirrhini) | 465010.03296 | best | - | - |
| (Anthropoidea,Tarsiidae,Strepsirrhini) | 465030.88379 | 20.85084 | 0.011* | 0.009* |
| (Anthropoidea,(Tarsiidae,Strepsirrhini)) | 465015.87140 | 5.83844 | 0.577 | 0.455 |
| ((Anthropoidea,Strepsirrhini),Tarsiidae) | 465014.28742 | 4.25447 | 0.720 | 0.534 |
|  |  |  |  |  |
| **Alternative positions of *Perodicticus*** |  |  |  |  |
| ((*Loris*,*Nycticebus*),(*Perodicticus*,Galagidae)) | 465010.03296 | best | - | - |
| ((*Loris*,*Nycticebus*),*Perodicticus*,Galagidae) | 465040.97399 | 30.94103 | 0.002* | 0.001* |
| (((*Loris*,*Nycticebus*),Galagidae),*Perodicticus*) | 465017.83206 | 7.79910 | 0.602 | 0.432 |
| (((*Loris*,*Nycticebus*),*Perodicticus*),Galagidae) | 465025.70414 | 15.67119 | 0.211 | 0.168 |
|  |  |  |  |  |
| **Alternative relationships among Lemuriformes** |  |  |  |  |
| (((Indriidae,Lepilemuridae),Lemuridae),Cheiroglaeidae) | 465010.03296 | best | - | - |
| (Indriidae,Lepilemuridae,Lemuridae,Cheiroglaeidae) | 465099.01739 | 88.98443 | <0.000* | <0.000* |
| ((Lemuridae,Indriidae),(Lepilemuridae,Cheiroglaeidae)) | 465040.18350 | 30.15055 | 0.149 | 0.140 |
| ((Lemuridae,Cheiroglaeidae),(Indriidae,Lepilemuridae)) | 465073.04533 | 63.01238 | <0.000* | <0.000* |
| ((Lemuridae,Lepilemuridae),(Indriidae,Cheirogaleidae)) | 465084.52363 | 74.49067 | 0.001* | 0.001* |
| (((Lemuridae,Indriidae),Lepilemuridae),Cheirogaleidae) | 465012.06899 | 2.03604 | 0.819 | 0.843 |
| (((Lemuridae,Indriidae),Cheiroglaeidae),Lepilemuridae) | 465067.69901 | 57.66605 | 0.003* | 0.001* |
| (((Lemuridae,Lepilemuridae),Indriidae),Cheirogaleidae) | 465019.15674 | 9.12378 | 0.232 | 0.633 |
| (((Lemuridae,Lepilemuridae),Cheiroglaeidae),Indriidae) | 465072.31325 | 62.28029 | 0.002* | 0.001* |
| (((Lemuridae,Cheiroglaeidae),Indriidae),Lepilemuridae) | 465084.88888 | 74.85592 | <0.000* | <0.000* |
| (((Lemuridae,Cheiroglaeidae),Lepilemuridae),Indriidae) | 465072.19636 | 62.16340 | 0.003* | 0.001* |
| (((Indriidae,Lepilemuridae),Cheirogaleidae),Lemuridae) | 465068.34300 | 58.31004 | 0.001* | 0.002* |
| (((Cheirogaleidae,Lepilemuridae),Indriidae),Lemuridae) | 465048.42966 | 38.39670 | 0.072 | 0.059 |
| (((Cheirogaleidae,Lepilemuridae),Lemuridae),Indriidae) | 465045.19217 | 35.15921 | 0.108 | 0.084 |
| (((Indriidae,Cheiroglaeidae),Lepilemuridae),Lemuridae) | 465078.13005 | 68.09710 | 0.001* | 0.001* |
| (((Indriidae,Cheiroglaeidae),Lemuridae),Lepilemuridae) | 465095.15364 | 85.12068 | <0.000* | <0.000* |
|  |  |  |  |  |
| **Alternative positions of Aotidae** |  |  |  |  |
| ((Callithrichidae,Aotidae),Cebidae) | 465010.03296 | best | - | - |
| (Callithrichidae,Aotidae,Cebidae) | 465021.62947 | 11.59651 | 0.048* | 0.046* |
| ((Callithrichidae,Cebidae),Aotidae) | 465019.86769 | 9.83473 | 0.108 | 0.096 |
| (Callithrichidae,(Cebidae,Aotidae)) | 465013.34901 | 3.31605 | 0.660 | 0.450 |
|  |  |  |  |  |
| **Alternative positions of *Saguinus*** |  |  |  |  |
| ((*Callithrix*,*Cebuella*),(*Saguinus*,*Leontopithecus*)) | 465006.85345 | best | - | - |
| (((*Callithrix*,*Cebuella*),*Saguinus*),*Leontopithecus*) | 465010.03296 | 3.17951 | 0.796 | 0.556 |
| ((*Callithrix*,*Cebuella*),*Saguinus*,*Leontopithecus*) | 465031.43578 | 24.5823 | 0.002* | 0.002* |
| (((*Callithrix*,*Cebuella*),*Leontopithecus*),*Saguinus*) | 465020.20380 | 13.35036 | 0.199 | 0.173 |
|  |  |  |  |  |
| **Alternative positions of *Mandrillus/Cercocebus*** |  |  |  |  |
| ((*Macaca*,(*Mandrillus*,*Cercocebus*)),((*Papio*,*Theropithecus*),*Lophocebus*)) | 465010.03296 | best | - | - |
| (*Macaca*,(*Mandrillus*,*Cercocebus*),((*Papio*,*Theropithecus*),*Lophocebus*)) | 465042.29678 | 32.26382 | 0.007* | 0.006* |
| (*Macaca*,((*Mandrillus*,*Cercocebus*),((*Papio*,*Theropithecus*),*Lophocebus*))) | 465030.38899 | 20.35603 | 0.134 | 0.080 |
| ((*Mandrillus*,*Cercocebus*),(*Macaca*,((*Papio*,*Theropithecus*),*Lophocebus*))) | 465031.65135 | 21.61839 | 0.097 | 0.058 |
|  |  |  |  |  |
| **Alternative positions of *Cercopithecus*** |  |  |  |  |
| ((*Erythrocebus*,*Cercopithecus*),*Chlorocebus*) | 465010.03296 | best | - | - |
| (*Erythrocebus*,*Cercopithecus*,*Chlorocebus*) | 465045.04022 | 35.00726 | 0.001* | <0.000* |
| ((*Chlorocebus*,*Cercopithecus*),*Erythrocebus*) | 465037.60442 | 27.57146 | 0.022* | 0.015* |
| ((*Chlorocebus*,*Erythrocebus*),*Cercopithecus*) | 465028.73099 | 18.69804 | 0.130 | 0.068 |
|  |  |  |  |  |
| **Alternative positions of *Semnopithecus*** |  |  |  |  |
| ((((*Nasalis,Simias*,*Pygathrix*),*Rhinopithecus*),*Semnopithecus*),(*Trachypithecus*,*Presbytis*)) | 465010.03296 | best | - | - |
| (((*Nasalis*,*Simias*,*Pygathrix*),*Rhinopithecus*),*Semnopithecus*,(*Trachypithecus*,*Presbytis*)) | 465023.60218 | 13.56922 | 0.041* | 0.029* |
| ((((*Nasalis*,*Simias*,*Pygathrix*),*Rhinopithecus*),(*Trachypithecus*,*Presbytis*)),*Semnopithecus*) | 465019.97838 | 9.94542 | 0.198 | 0.128 |
| (((*Nasalis*,*Simias*,*Pygathrix*),*Rhinopithecus*),((*Trachypithecus*,*Presbytis*),*Semnopithecus*)) | 465018.03249 | 7.99953 | 0.342 | 0.214 |

* P<0.05
