# Supplementary figures and images for: A Mitogenomic Phylogeny of Living Primates
Source: PLoS One. 2013 Jul 16;8(7):e69504. doi: 10.1371/journal.pone.0069504 (PMC3713065; doi:10.1371/journal.pone.0069504)

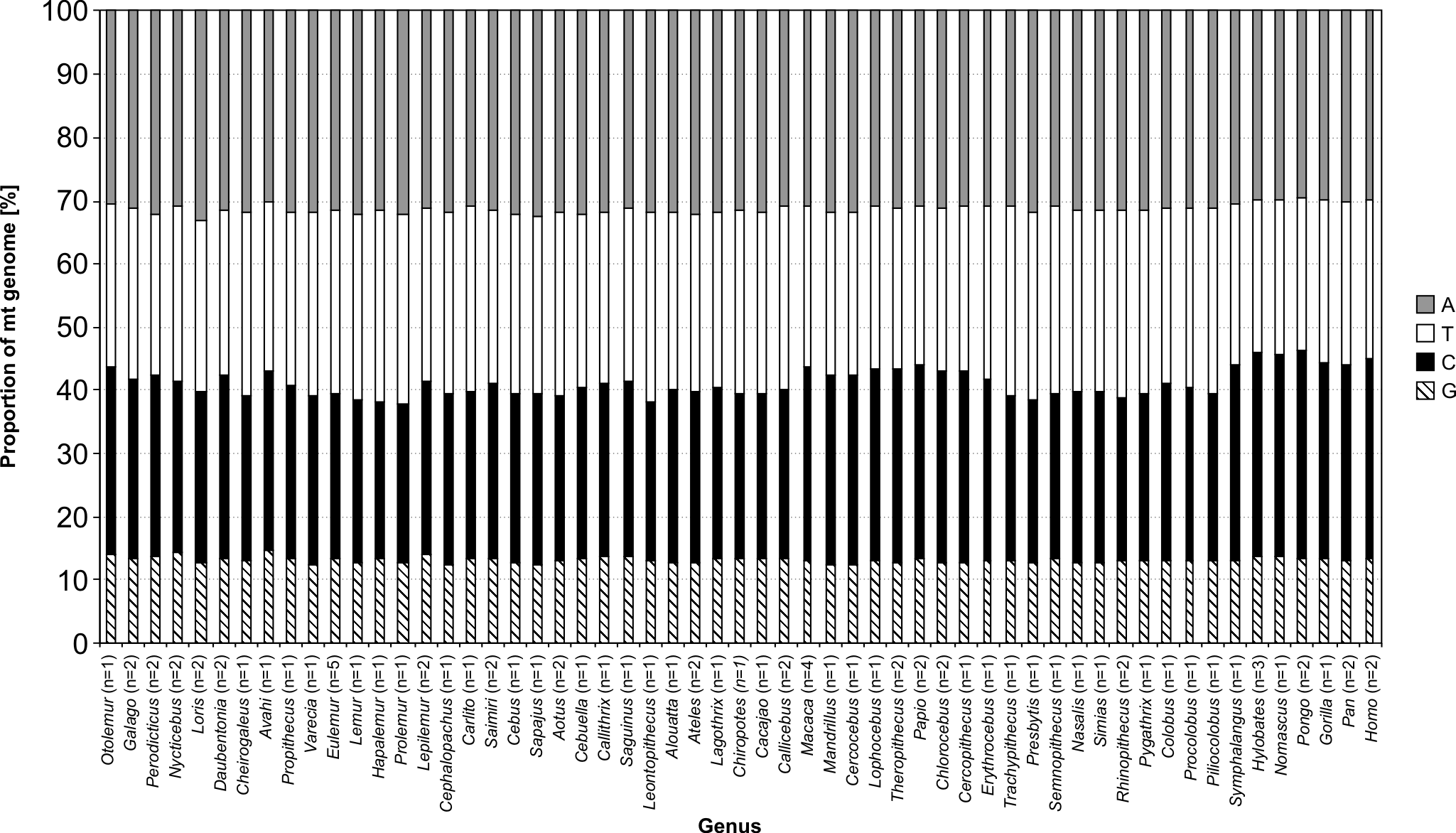

Supplement: Figure S1 — Diagram showing the G/C content of the mt genomes of the studied genera. (TIF) [file pone.0069504.s006.tif]

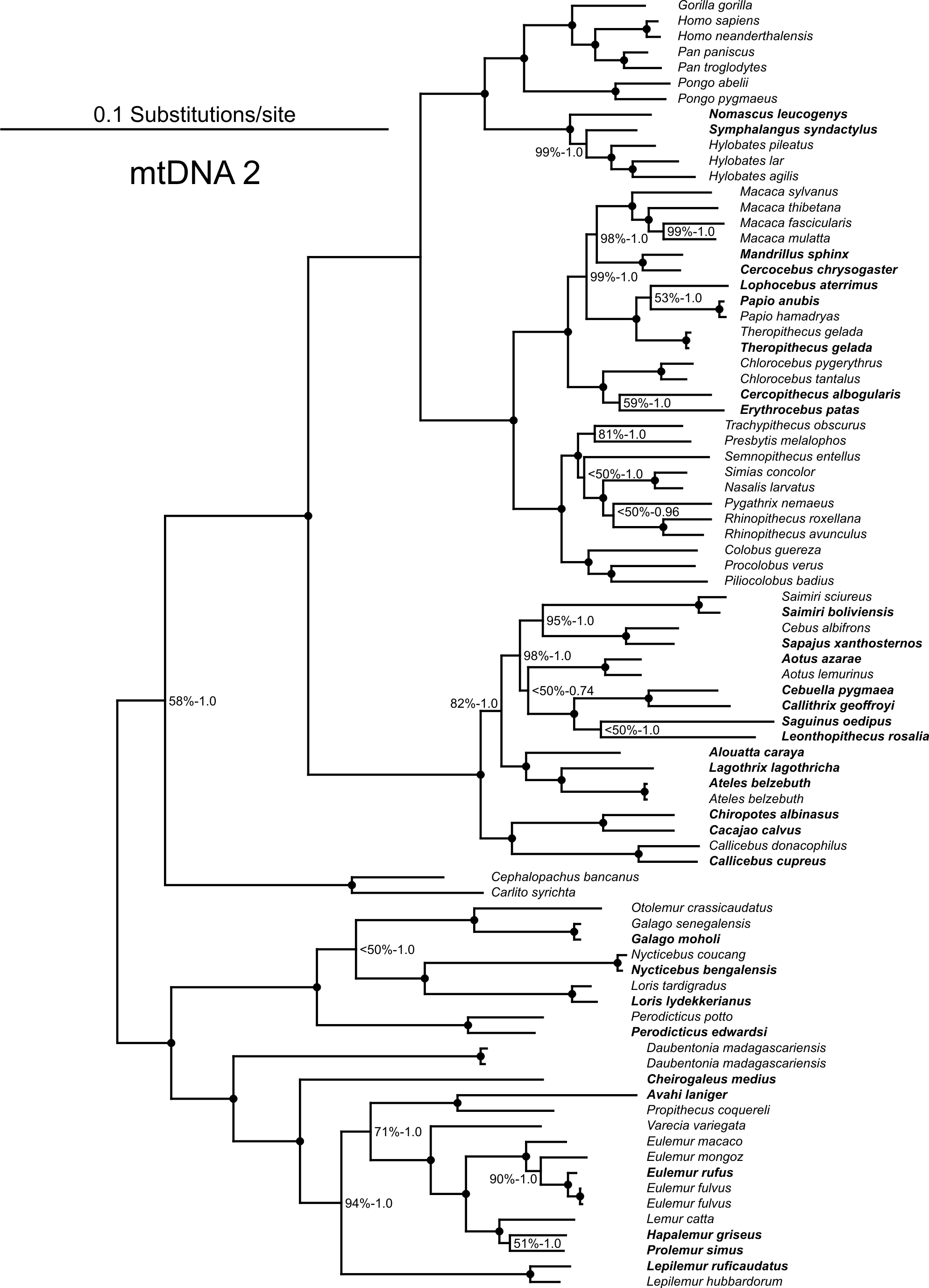

Supplement: Figure S2 — Phylogram as obtained from dataset mtDNA2. Newly generated sequences are indicated in bold. Black dots on nodes indicate ML support and Bayesian posterior probabilities of 100% and 1.0, respectively. Values below are shown at the respective branches. (TIF) [file pone.0069504.s007.tif]

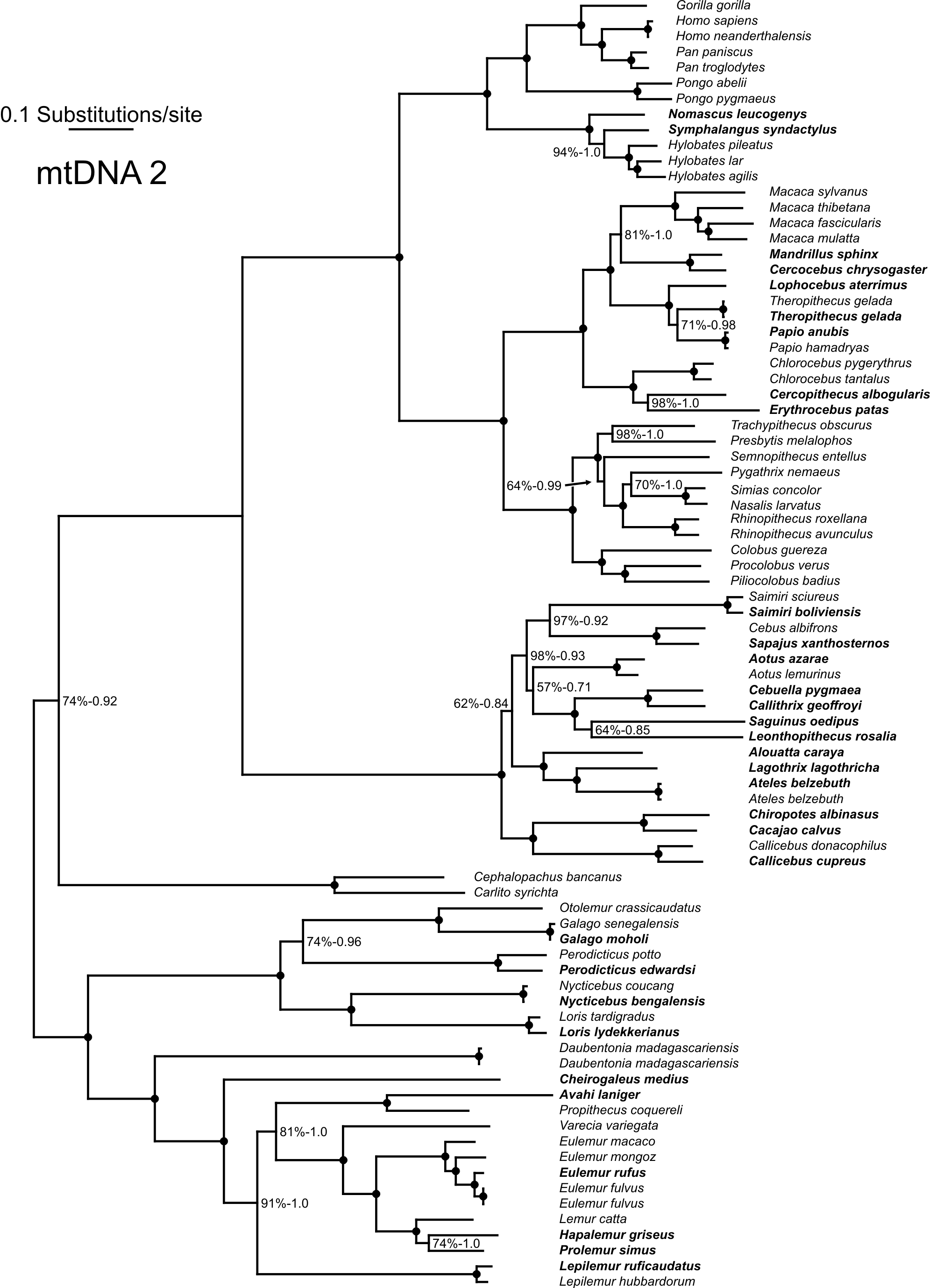

Supplement: Figure S3 — Phylogram as obtained from dataset mtDNA3. Newly generated sequences are indicated in bold. Black dots on nodes indicate ML support and Bayesian posterior probabilities of 100% and 1.0, respectively. Values below are shown at the respective branches. (TIF) [file pone.0069504.s008.tif]

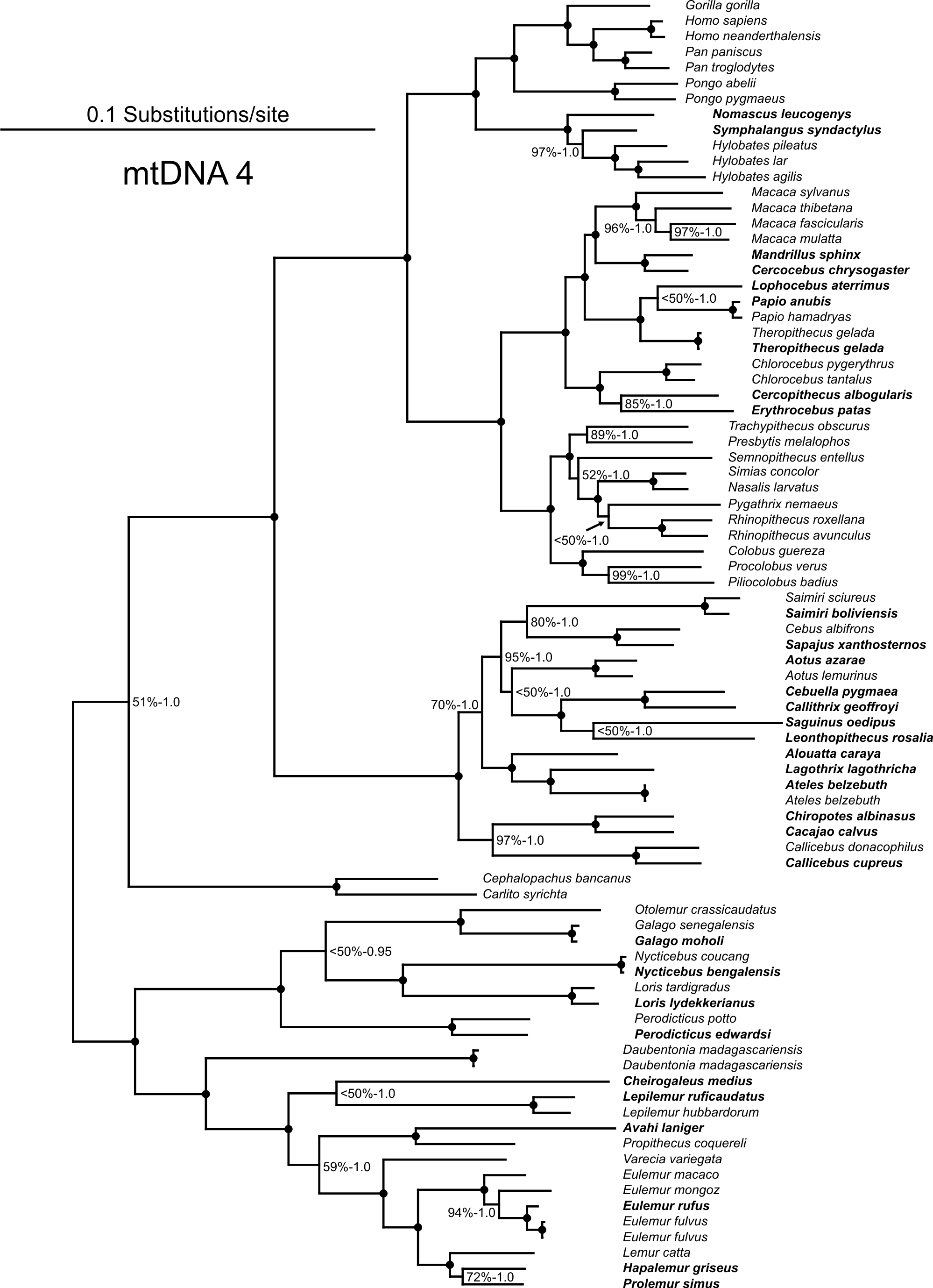

Supplement: Figure S4 — Phylogram as obtained from dataset mtDNA4. Newly generated sequences are indicated in bold. Black dots on nodes indicate ML support and Bayesian posterior probabilities of 100% and 1.0, respectively. Values below are shown at the respective branches. (TIF) [file pone.0069504.s009.tif]
